# Remitting seronegative symmetrical synovitis with pitting edema: a case report

**DOI:** 10.1186/s13256-022-03310-0

**Published:** 2022-03-24

**Authors:** Yasushi Tanaka, Kohki Kohchi, Kazuhiro Kitamoto

**Affiliations:** Present Address: Department of General Internal Medicine, Yodogawa Christian Hospital, 1-7-50 Kunijima Higashiyodogawa, Osaka, Japan

**Keywords:** RS3PE, Refractory edema, Symmetrical

## Abstract

**Background:**

Edema occurs in various disorders. One of those is remitting seronegative symmetrical synovitis with pitting edema, a rare syndrome whose pathophysiology is not clearly understood. We report herein a case of refractory edema diagnosed as remitting seronegative symmetrical synovitis with pitting edema.

**Case presentation:**

A 82-year-old Asian male was admitted to the Department of General Internal Medicine with a 2-month history of symmetrical swelling of both hands. Despite treatment with loop diuretic furosemide 40 mg daily, his condition did not respond to the medication and his quality of life deteriorated. An examination of the joints showed tenderness suggestive of synovitis with restricted movements in bilateral proximal interphalangeal joint. Laboratory findings revealed hyperglycemia, elevated erythrocyte sedimentation rate 118 mm/hour, and elevated C-reactive protein 6.58 mg/dL. Plain radiographs of both hands showed soft tissue swelling, changes consistent with osteoarthritis, and no erosions. The diagnosis of bilateral remitting seronegative symmetrical synovitis with pitting edema was made. Treatment with prednisolone 15 mg daily was instituted.

**Conclusions:**

Although remitting seronegative symmetrical synovitis with pitting edema is rare, it should be remembered as a disease that causes edema in the elderly.

## Introduction

Edema including in the dorsum of hands occurs in various disorders, such as heart failure, renal failure, cirrhosis, hypothyroidism, venous or lymphatic abnormality, inflammatory arthritis, and cancer. Conventional treatment includes restricting dietary sodium and using diuretics, usually loop diuretics, accompanied by specific treatments for each clinical disorder [1]. Meanwhile, remitting seronegative symmetrical synovitis with pitting edema (RS3PE) is a rare syndrome whose pathophysiology is not clearly understood. High inflammatory markers, negative rheumatoid factor (RF), and prompt response to low-dose steroids are hallmarks of the disease. This article presents a case of refractory edema diagnosed as RS3PE followed by a brief review of literature.

## Case presentation

**Fig. 1:**
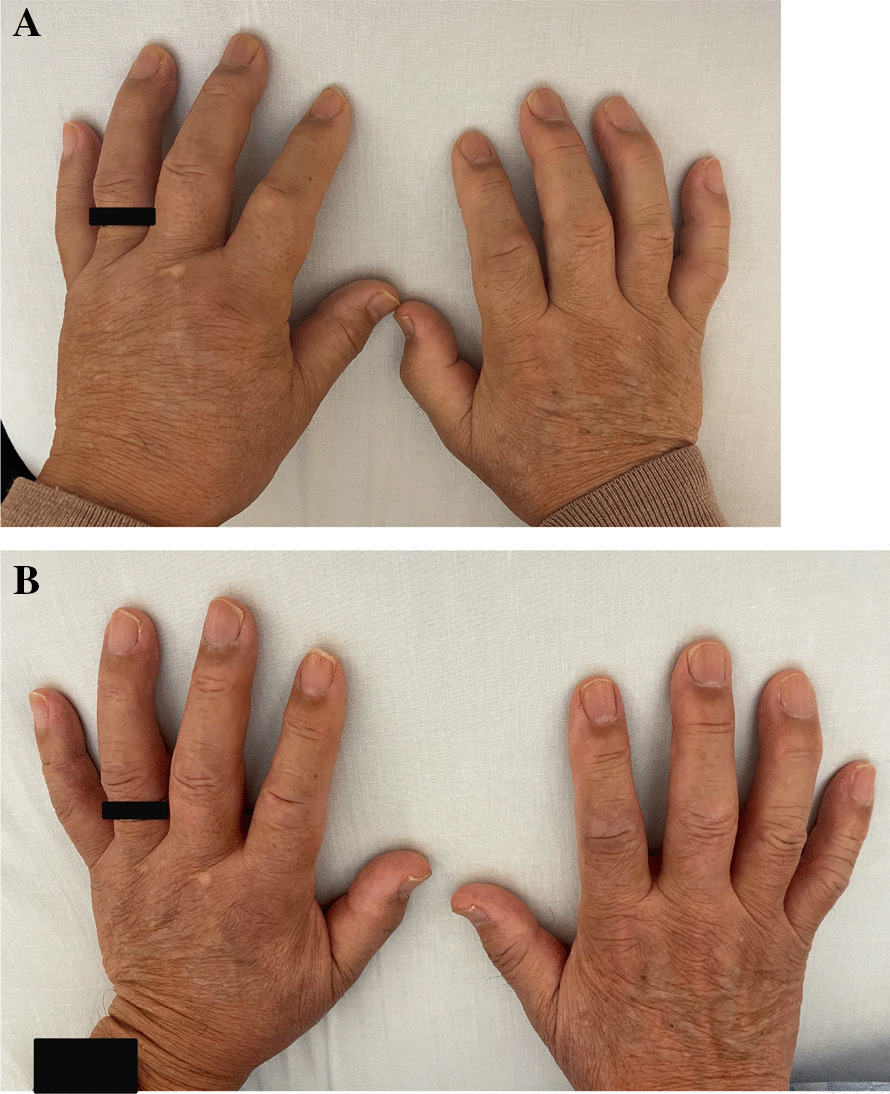
**A** Symmetrical pitting edema of dorsum of both hands. **B** Swelling on dorsum of hands drastically resolved

A 82-year-old Asian male was admitted to the Department of General Internal Medicine with 2-month history of symmetrical swelling in both hands. The swelling was localized to the dorsum of hands and did not extend to the face and lower extremities. He had no dyspnea on exertion and orthopnea. He had good appetite, and there was no significant history of weight loss. He denied any history of jaundice in the past. Despite treatment with loop diuretics furosemide 40 mg daily, his condition did not respond to the medication and his quality of life deteriorated. Therefore, he visited our hospital and requested an alternative treatment. On physical examination, his vitals were stable with blood pressure of 147/85 mmHg and heart rate of 98 beats per minute. He had swelling restricted to the dorsum of both hands with pitting edema (Fig. 1A). Examination of the joints showed tenderness suggestive of synovitis with restricted movements in bilateral proximal interphalangeal joint. Other clinical examinations including cardiovascular, respiratory, abdominal, and neurological examinations were unremarkable.

Laboratory findings revealed hyperglycemia, elevated erythrocyte sedimentation rate (ESR) of 118 mm/hour, and elevated C-reactive protein (CRP) of 6.58 mg/dL, and slight anemia with hemoglobin of 12.1 g/dL (Table 1). Plain radiographs of both hands showed soft tissue swelling with neither narrowing of the joint spaces nor bone erosion. At this point, the diagnosis of bilateral RS3PE was made. Treatment with prednisolone 15 mg daily was instituted. At follow-up after hospitalization, dramatic improvement of all his symptoms was noted. The swelling on the dorsum of his hands had drastically resolved (Fig. 1B). At 1 week follow-up after hospitalization, ESR and CRP had returned to normal. He was discharged on hospital day 12 and continues to be followed up on an outpatient basis. He remained symptom free at 2-week review on prednisolone 15 mg daily, which was then reduced to 10 mg daily. Thereafter, the prednisolone dose was reduced by 1 mg every 4 weeks, and he has progressed without any flare-up of inflammation.

**Table 1:** Laboratory data on admission

Parameter	Recorded value	Standard value
White blood cell count	89 × 10^2^/μL	32–85 × 10^2^/μL
Neutrophils	78.2%	
Lymphocytes	17.0%	
Eosinophils	0.4%	
Hemoglobin	12.1 g/dL	11.3–15.2 g/dL
Hematocrit	36.1%	36–45%
Platelets	29.2 × 10^4^/μL	13–34.9 × 10^4^/μL
C-reactive protein	6.58 mg/dL	≤ 0.29 mg/dL
Erythrocyte sedimentation rate	118 mm/hour	1–10 mm/hour
Total protein	6.9 g/dL	6.5–8 g/dL
Albumin	3.2 g/dL	4–5.2 g/dL
Aspartate aminotransferase	19 U/L	0–30 U/L
Alanine aminotransferase	17 U/L	0–30 U/L
Lactate dehydrogenase	135 U/L	106–220 U/L
Creatine phosphokinase	36 U/L	62–287 U/L
Blood urea nitrogen	13.6 mg/dL	7–24 mg/dL
Creatinine	0.91 mg/dL	0–1 mg/dL
Sodium	139 mEq/L	136–147 mEq/L
Potassium	3.8 mEq/L	3.6–5.0 mEq/L
Glucose	131 mg/dL	70–99 mg/dL
Rheumatoid factor	Negative	
Anti-CCP antibody	Negative	

## Discussion and conclusion

In daily medical practice, physicians are often faced with patients with edema of unknown etiology. Edema can be localized or generalized. RS3PE is a rare autoimmune condition. The first description was in 1985 in a series of ten patients by McCarty *et al.* [2]. The patient presented herein satisfied all four of McCarty’s RS3PE diagnostic criteria of localized edema particularly in the dorsum of hands and/or legs, acute onset of polyarthritis, age greater than 50 years, and seronegativity for RF.

Mixed connective tissue disease and systemic sclerosis are the differentials for swelling of the hands with arthritis. However, there was no skin sclerosis or erythema in this case. Because of the short follow-up period, we could not exclude the association with malignancy. We could not exclude joint erosion as early changes because we did not perform ultrasonography or magnetic resonance imaging (MRI) of the wrist joint.

Corticosteroids are the mainstream of treatment for RS3PE. Typically, the starting dose is the steroid equivalent of prednisolone 15–20 mg daily. According to previously published cases, corticosteroids were sustained at this initial dose for 2–3 weeks then tapered over 12–18 months [3–5].

In general, edema of hands evidently reduces patient quality of life, as in this case.

## Data Availability

Not applicable.

## Abbreviations

RS3PE: Remitting seronegative symmetrical synovitis with pitting edema
RF: Rheumatoid factor
ESR: Erythrocyte sedimentation rate
CRP: C-reactive protein
